# Diversity analysis of MSP1 identifies conserved epitope organization in block 2 amidst high sequence variability in Indian *Plasmodium falciparum* isolates

**DOI:** 10.1186/s12936-018-2592-y

**Published:** 2018-12-03

**Authors:** Sharmistha Ghoshal, Pragya Gajendra, Sumana Datta Kanjilal, Mitashree Mitra, Sanghamitra Sengupta

**Affiliations:** 1 Department of Biochemistry, University of Calcutta, 35, Ballygunge Circular Road, Kolkata, West Bengal 700 019 India; 2 School of Studies in Anthropology, Pt. Ravishankar Shukla University, Raipur, Chhattisgarh 492010 India; 3 Department of Pediatric Medicine, Institute of Post Graduate Medical Education & Research, Kolkata, West Bengal India

**Keywords:** *Plasmodium falciparum*, MSP1, Genetic diversity, Multiclonal infection, Epitope organization, India

## Abstract

**Background:**

Despite its immunogenicity, the polymorphic nature of merozoite surface protein 1, an important vaccine candidate for *Plasmodium falciparum* malaria, remains a concern. This study analyses the impact of genetic variability and parasite population structure on epitope organization of different MSP1 segments.

**Methods:**

Altogether 98 blood samples collected from *P. falciparum* infected mild and severe malaria patients of Chhattisgarh and West Bengal were used to sequence regions encoding block 2 and MSP1-19 of *msp1*. Sequences were analysed using MEGA7, DnaSPv5, Arlequin3.5 and BepiPred.

**Results:**

All three major MSP1 block 2 allele families namely K1, MAD20 and RO33 were detected in the samples and they together resulted in 41 indel variants. Chhattisgarh samples displayed an average MOI of 2.07 ± 1.59 which was higher in mild malaria and in age group < 18 years. Ultra-structure of block 2 alleles revealed that mutation and repeat expansion were two major mechanisms responsible for allelic variability of K1 and MAD20. Regions flanking block 2 were highly variable in Chhattisgarh with average mismatch differences (k) ranging from 1.198 to 5.156 for three families. In contrast, region encompassing MSP1-19 exhibited limited heterogeneity (k_Chhattisgarh_ = 1.45, k_West Bengal_ = 1.363). Of the 50 possible B cell linear epitopes predicted from block 2 variants, 94.9% (131 of 138) of the parasites could be represented by three conserved antigens.

**Conclusions:**

Present data indicates that natural selection and transmission intensity jointly play a role in controlling allelic diversity of MSP1 in Indian parasite isolates. Despite remarkable genetic variability, a limited number of predominant and conserved epitopes are present in Indian parasite isolates reinstating the importance of MSP1 as a promising malaria vaccine candidate.

**Electronic supplementary material:**

The online version of this article (10.1186/s12936-018-2592-y) contains supplementary material, which is available to authorized users.

## Background

The estimated rate of malaria mortality has reduced by 47% worldwide between 2000 and 2013 [1]. This reduction of malaria burden has been achieved through coordinated control of parasites and vectors using a variety of interventions [2]. To sustain this encouraging statistics and prevent clinical disease in sub-Saharan Africa, Asia and Latin America which continue to share a disproportionately high global malaria load, development of vaccine against the most virulent species, *Plasmodium falciparum*, in particular, is urgently needed [3]. Till date RTS, S remains the most advanced malaria vaccine, although its mechanism action and factors responsible for inter-individual differences in vaccine efficacy are poorly characterized [4]. Of the different vaccine development strategies, those targeting pre-erythrocytic stage proteins and asexual blood stage antigens are primarily intended to prevent clinical disease. However, many blood stage merozoite proteins that elicit protective immunity against malaria use parallel redundant pathways and/or are extremely polymorphic [5, 6]. A polymorphic antigen with strong immunogenicity may still be considered as the component of a multistage polyvalent vaccine and protect the vulnerable populations in diverse transmission settings. As a proof of concept, a synthetic vaccine was constructed by fusing block 2 variants with conserved block 1 of *P. falciparum* merozoite surface protein 1. This hybrid vaccine produced high titre antibodies in experimental animals inhibiting parasite growth in vitro and showed strong reactivity against antibodies isolated from naturally exposed malaria patients in a Ghanaian cohort [7].

MSP1 is the most abundant surface antigen in the blood stage of *P. falciparum*. It plays a crucial role in the initial low affinity attachment of parasite to RBC membrane during erythrocyte invasion [8]. MSP1 contains 17 blocks of which block 2 shows extensive allelic polymorphism worldwide [9, 10]. Block 2 alleles are mainly represented by three families namely K1, MAD20 and RO33 in the field isolates based on their characteristic tri-peptide motifs. Different allelic sequences belonging to these families show highly skewed and continent specific geographical distribution [11]. Besides, the pattern and extent of fragment size polymorphism of block 2 alleles serve as molecular indicators host immunity and malaria transmission dynamics [12].

MSP1 is synthesized as a ~ 195 kDa precursor which is proteolytically cleaved into four major fragments prior to schizont rupture [13]. One of these fragments, MSP1-42 is further processed to produce MSP1-19 that enters with merozoite into RBCs whereas others are shed off [14]. MSP1-19 is immunogenic in both human and animal infections and is considered as an attractive vaccine candidate [15–21]. Studies evaluating the immunogenic potential of the rest of the MSP1 molecule identified block 2 region as a target of protective immunity and showed that antibodies to block 2 are also associated with reduced risk of clinical malaria [7, 22, 23].

Given this, the present study evaluates the genetic diversity of two most immunogenic segments of *msp1* namely block 2 and MSP1-19 in parasite isolates from Chhattisgarh, in central India and West Bengal, in eastern India. In parallel, the question, how the observed allelic variation of these segments affects the distribution of B-cell epitopes, is also addressed. The results indicate that *msp1* block 2 gene pool is shaped by a localized pattern of parasite transmission and its immunogenic repertoire is furnished with a limited number of conserved epitopes. The suitability of the MSP1 block 2 as a potential vaccine target, as revealed by the present report, may have significant implications in the global malaria eradication initiatives.

## Methods

### Sample collection and DNA analysis

Peripheral blood samples for parasite DNA analysis were collected from Ambikapur situated in Chhattisgarh, central India, during a period of 2010–2013. Owing to its distinct ecological and geographical conditions, malaria exhibits a discrete pattern in Chhattisgarh contributing to ~ 12% of total disease burden and the highest share of deaths (17%) in India [24]. Genomic DNA extracted from peripheral blood samples of *P. falciparum* malaria patients admitted in Calcutta National Medical College & Hospital, Kolkata, in the year 2010 were also included in the study [25]. Kolkata is the capital of West Bengal which accounts for about 10% of the total malaria cases in India [26]. The two study regions differ with regard to malaria transmission intensity and disease characteristics [27, 28].

Peripheral blood samples collected from *P. falciparum* malaria patients of Ambikapur were employed to isolate genomic DNA using QIAamp DNA Blood Midi Kit (Qiagen, Hilden, Germany) following manufacturer’s protocol. Overall, 98 *P. falciparum* infected blood samples, 41 from Chhattisgarh and 57 from West Bengal, detected through Giemsa-stained thick and thin smears, were selected for this study. Patients suffering from co-infection with *Plasmodium vivax* were excluded from the analysis. In addition, patients with acute lower respiratory tract infection, bacteraemia, measles, severe diarrhoea with dehydration and other chronic or severe conditions, such as cardiac, renal or hepatic diseases, AIDS, G6PD deficiency, sickle cell anaemia, typhoid and cancer were also excluded.

### PCR amplification and cloning of PCR amplicons

**Fig. 1:**
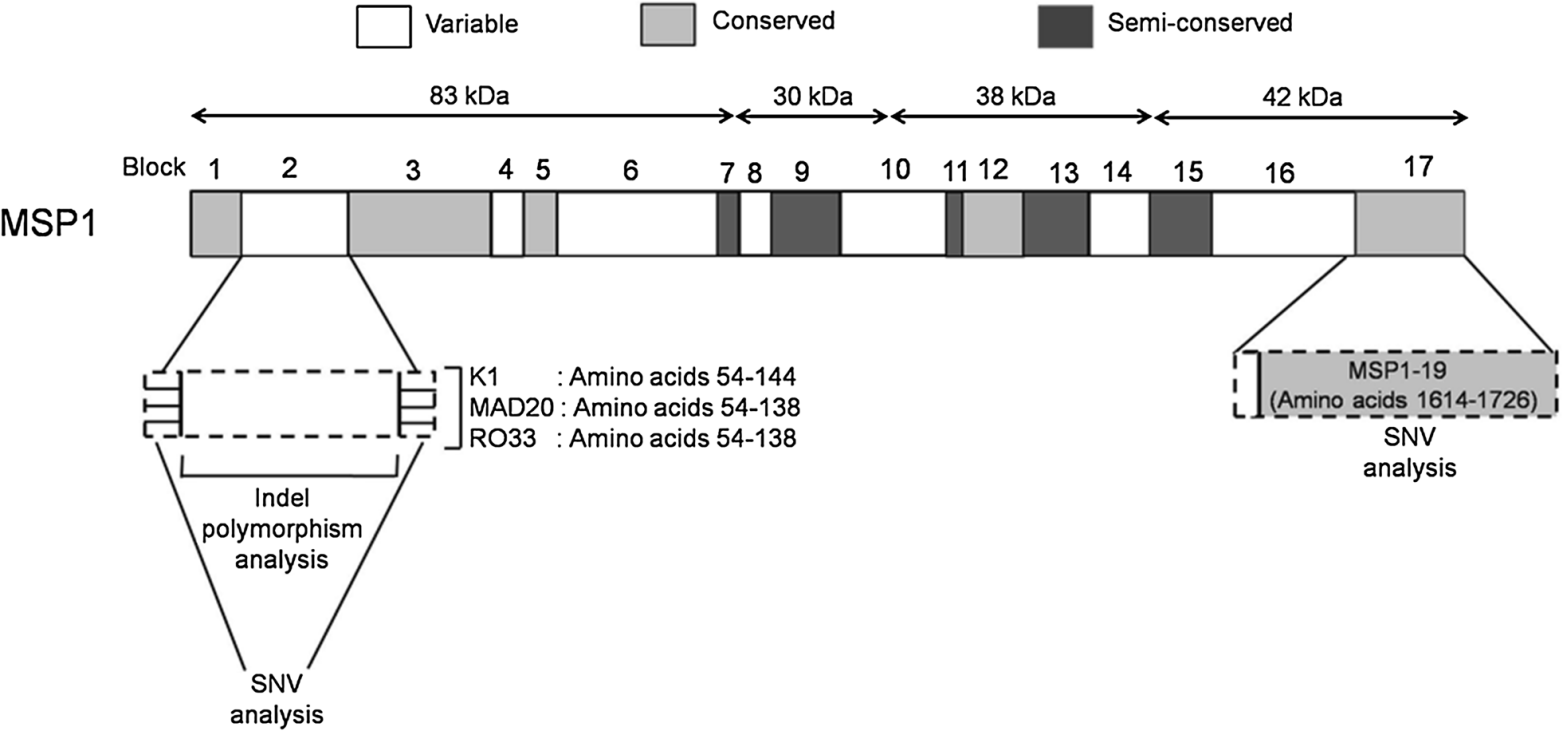
Schematic representation of *P. falciparum* merozoite surface protein 1 (MSP1). The regions subjected to sequence analysis were highlighted using broken lines

Oligonucleotide primers were designed for each target region using *P. falciparum* genomic DNA sequence (3D7 strain: GenBank accession number U65407.1) (Fig. 1). Primers were designed from the conserved sequences located on the both end of hypervariable block 2 (MSP1 block 2 forward: 5′-CACATGAAAGTTATCAAGAACTTGTC-3′, MSP1 block 2 reverse: 5′-TAAGTACGTCTAATTCATTTGCACG-3′) [29]. Region encoding the receptor binding site of MSP1 (MSP1-19) was also PCR amplified using MSP1-19 forward: 5′-CGTCACCAGCAAAAACAGACGAAC-3′ and MSP1-19 reverse: 5′-TGCTACCTGAATCTTCTTCGGTAC-3′ primers. Amplification of both target regions was performed in 15 μL reaction mixtures containing 0.2 mM dNTP, 1.5 mM MgCl_2_, 0.4 μM of each primer, and 1 U of GoTaq^®^ Flexi DNA polymerase (Promega). The cycling conditions for PCR consisted of an initial denaturation at 94 °C for 5 min, followed by 35 cycles of denaturation at 94 °C for 45 s, annealing at 58 °C for 45 s, extension at 72 °C for 45 s, and a final extension at 72° C for 5 min using a thermal cycler (Applied Biosystems^®^ GeneAmp^®^ PCR System 9700). The amplicons were visualized using UV transillumination on gel documentation system (Biostep) following electrophoresis on 2% agarose gel (Promega). PCR products showing single band were purified by Qiaquick gel extraction kit (QIAGEN India Pvt. Ltd, Hilden, Germany) and sequenced. PCR amplicons showing more than one band in gel electrophoresis images were suspected to represent multiclonal infections. To analyse those samples, each PCR product showing multiple bands was cloned in pTZ57R/T vector using InsTAclone PCR Cloning Kit (Fermentas) and transformed into DH5α *Escherichia coli* strain. Transformed *E. coli* were cultured on Luria–Bertani Agar containing 100 μg/μL Ampicillin. Ten colonies were chosen arbitrarily for each PCR amplicon to isolate the plasmids. Altogether, 16 samples showing multiple bands were analysed.

### Sequencing

Purified PCR products showing single band and each of the isolated plasmids containing single genotype were sequenced using the same primers used in PCR. Sequencing PCR protocol was programmed with an initial denaturation at 94 °C for 30 s followed by 25 cycles (or 29 cycles, while plasmid DNA was used as templates) of denaturation at 94 °C for 10 s, holding for 10 s at 50 °C and extension for 4 min at 60 °C and finally stored at 4 °C. Sequencing was carried out in both directions, using the forward and reverse primers and Big Dye v3.1 dye terminator chemistry. The products were resolved on ABI Prism 3100 Genetic Analyzer (Applied Biosystems, Foster City, CA).

### Sequence alignment and data analysis

Raw sequence data files from field isolates were manually revised to exclude signal noises. To compare the sequence identity, NCBI BLAST analysis was performed with all test sequences [30, 31]. Nucleotide sequences generated were submitted to the GenBank database under accession numbers MF772523–MF772713. MEGA7 tool was used to perform multiple sequence alignment and to translate DNA sequences into amino acid codes. Allele specific sequence motifs were used to search MSP1 block 2 sequences to assign family types. Sequences belonging to a given family were clustered to detect the pattern of fragment length polymorphism [32]. Based on nucleotide sequences pertaining to block 2, phylogenetic tree representing each allele family was constructed using maximum Parsimony method (MEGA7). This assisted further sub-classification of each allele types.

Association of allele frequency with transmission intensity, disease severity and multiclonality were examined using Chi square statistics while between group comparisons of multiplicity of infection (MOI) were conducted using Student’s t-test [33]. A p value < 0.05 was considered to be statistically significant. Single nucleotide variations (SNVs) were used to estimate several genetic diversity parameters using DnaSPv5 [34, 35]. These included (i) number of segregating sites (S), (ii) average number of pairwise nucleotide differences within population (k), (iii) average number of observed nucleotide differences per site between any two sequences (π), (iv) Watterson’s θ (θw). Estimation of Tajima’s D, Fu & Li’s statistics and the minimum number of recombination event (Rm) in regions corresponding to MSP1 block 2 and MSP1-19 was carried out using DnaSPv5. Tajima’s D and Fu & Li’s statistics were used to assess the neutral theory of evolution. The significance of Tajima’s *D* statistics was indicated by its confidence limits while that of Fu and Li’s D* and F* statistics were represented by its critical values [36–38]. The intra- and inter-population genetic differentiation were measured by the fixation index (F_ST_) using the Arlequin software package version 3.5 [39, 40].

### Prediction of B-cell linear epitopes for block 2 and MSP1-19 allelic variants

Linear B-cell epitopes were predicted from MSP1 block 2 and MSP1-19 amino acid sequences using BepiPred [41]. BepiPred combines predictions of a hidden Markov model and a propensity scale method developed by Parker et al. [42, 43]. It analyses each amino acid independently to assign a score between − 3 and 3. The strength of prediction by BepiPred is defined in terms of sensitivity and specificity. On the basis of a benchmark calculation containing 85 B-cell epitopes, dependence of sensitivity and specificity of BepiPred at different selected thresholds was estimated (Additional file 1: Table S1) [44]. In this study, analysis of epitopes was conducted using two different threshold scores namely 0.35 and 1.30. The threshold score of 0.35 was chosen since at this score the sensitivity and specificity estimates were optimum. A stringent threshold of 1.3 was chosen to improve the strength of prediction by maximizing the specificity feature. A minimum of 7 consecutive residues each displaying a score above the specified threshold was considered to be an epitope.

## Results

### Indel polymorphism of MSP1 block 2 and multiplicity of infection

**Fig. 2:**
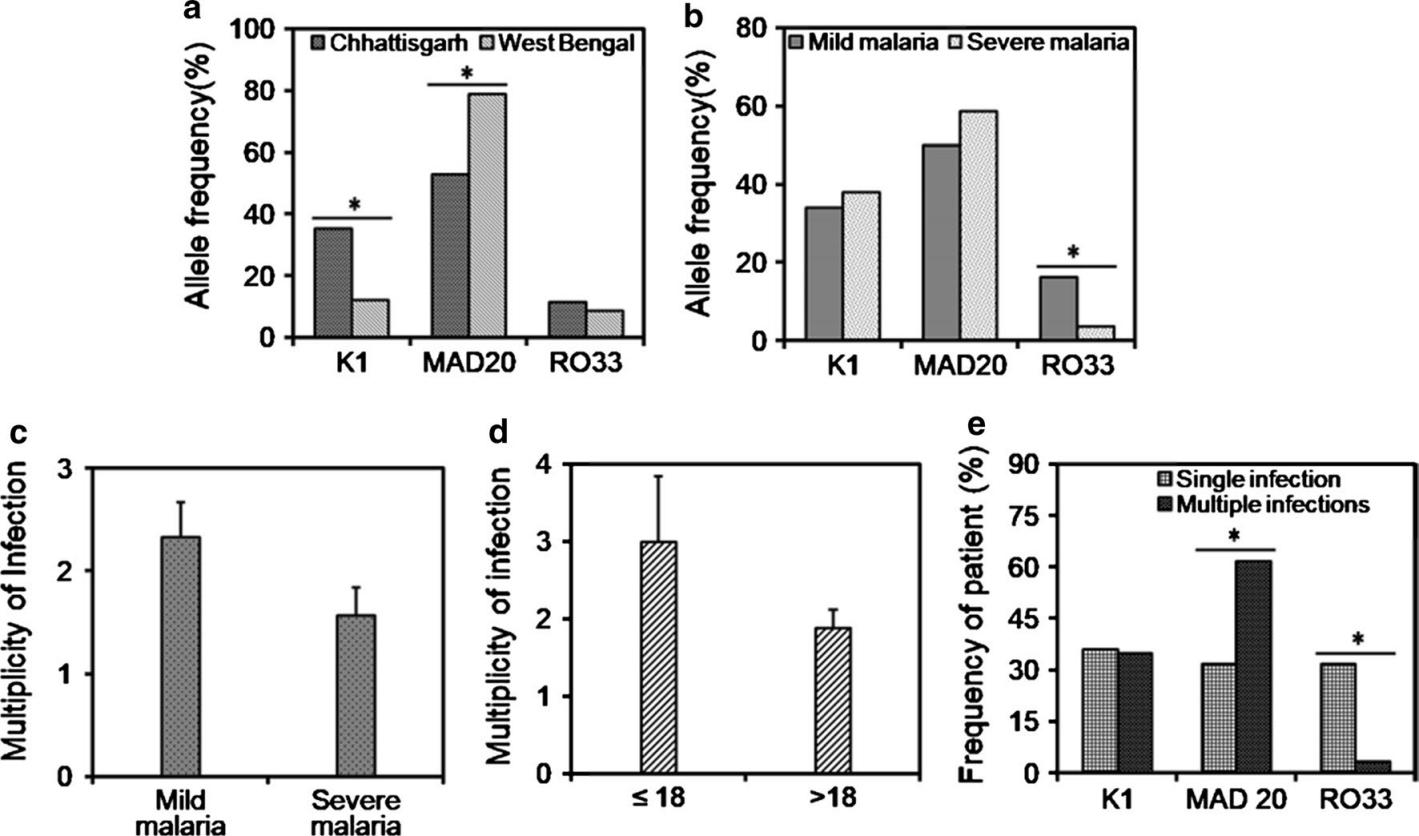
Analysis of frequencies of *msp1* block 2 alleles and multiplicity of infections in different groups. **a** Frequencies of *msp1* alleles in Chhattisgarh and West Bengal. **b** Distribution of *msp1* alleles in Chhattisgarh patients with mild and severe malaria. **c** Comparison of MOI in the mild and severe malaria patients of Chhattisgarh. **d** Differences of MOI in two different age groups of Chhattisgarh patients. **e** Frequencies of K1, MAD20, RO33 alleles associated with single and multiple infections in Chhattisgarh. Asterisk indicates p < 0.05 in Chi square test

A total of 98 malaria patients (41 from Chhattisgarh and 57 from West Bengal) were employed for the genetic analysis of *msp1* block 2 of *P. falciparum*. All three major allelic families namely K1, MAD20 and RO33 were detected in Chhattisgarh and West Bengal with frequencies of K1 (χ^2^ = 14.7, p < 0.001) and MAD20 (χ^2^ = 16.1, p < 0.001) differing significantly between two study sites (Fig. 2a). Since the patients of West Bengal suffered from mild malaria, the correlation between MSP1 allelic varieties with severity of disease was examined in Chhattisgarh data only. Frequency of RO33 (χ^2^ = 9.83, p < 0.01) was significantly higher in mild infection (Fig. 2b). Keeping in line with the low transmission intensity of the region, multiclonal infections were not detected in West Bengal samples. On the other hand, 39.02% of Chhattisgarh patients suffered from multi-genotypic infections, resulting in a mean MOI of 2.07 ± 1.59. MOI was higher in patients with mild malaria (2.33 ± 1.78) than those with severe malaria (1.57 ± 1.02), although the difference was not statistically significant in two-tailed Student’s t test (Fig. 2c). To detect if there was any association of MOI with age, Chhattisgarh patients were classified into two age groups namely (i) ≤ 18 years (n = 7) and (ii) > 18 years (n = 34). MOI was higher in the patients below 18 years of age (≤ 18 years: 3 ± 2.24 and > 18 years: 1.88 ± 1.39 (Fig. 2d). A comparative analysis of distribution of the *msp1* allelic families between single and multiple infections showed a statistically significant prevalence of MAD20 (χ^2^ = 18.1, p < 0.001) in patients suffering from multi-genotype infections whereas RO33 (χ^2^ = 29.1, p < 0.001) predominated in single infection (Fig. 2e). Taken together, MAD20 displayed an extensive within and between population variation whereas K1 exhibited polymorphism only within Chhattisgarh patients.

**Fig. 3:**
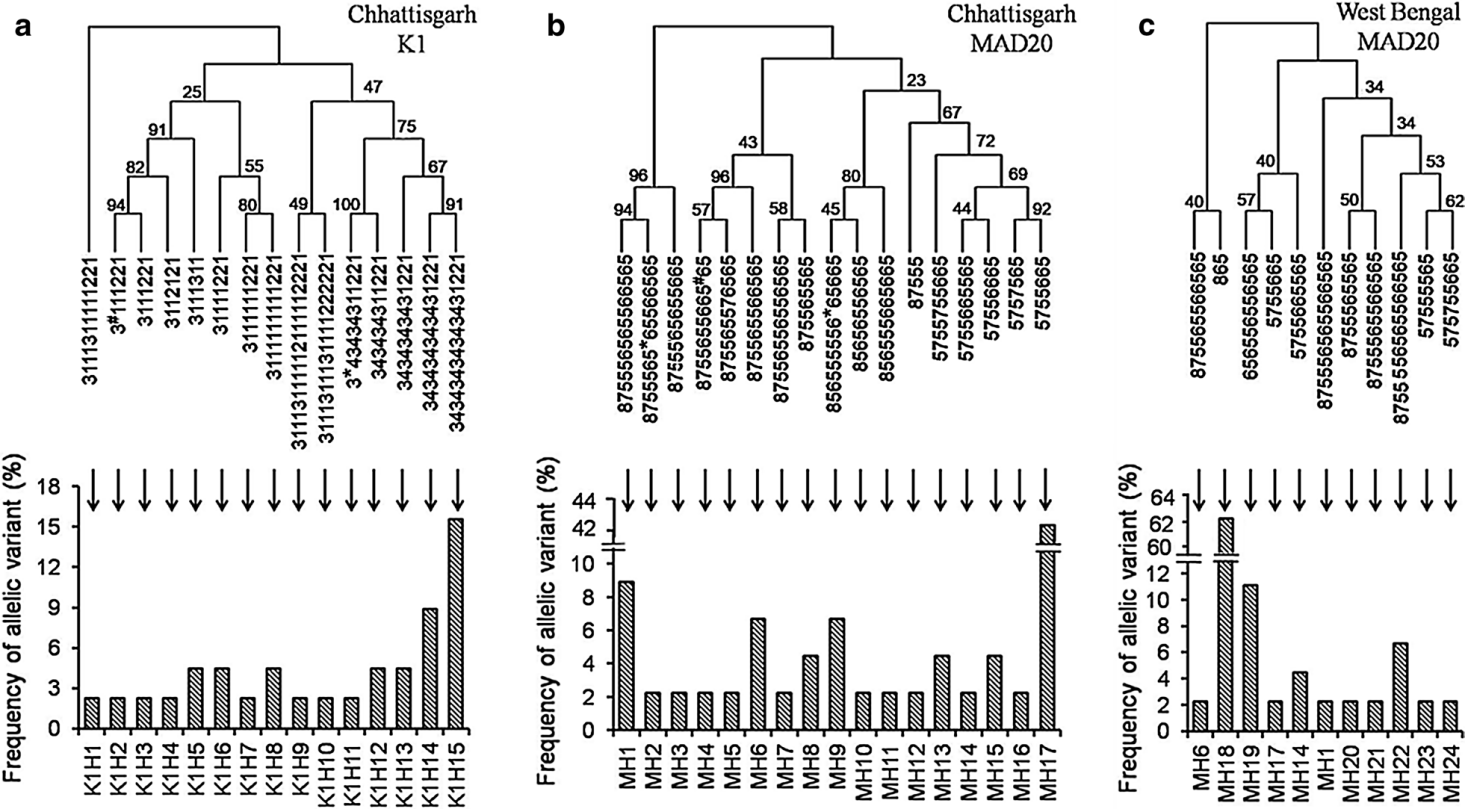
Phylogenetic relationship and prevalence of different *msp1* sub-alleles. **a** Organization of tri-peptide motifs in the alleles belonging to K1 family in Chhattisgarh parasite population and their respective proportions. **b** Organization and prevalence of tri-peptide motifs in the alleles belonging to MAD20 family in Chhattisgarh sample. **c** Organization and prevalence of tri-peptide motifs in the alleles belonging to MAD20 in West Bengal samples. Bootstrap values were shown for each branch of the Maximum Parsimony tree. SGT, SGP, SAQ, SGA, STQ and SAR repeats were present in K1 and denoted as 1, 2, 3, 4, 3* and 3^#^, respectively and SGG, SVA, SVT, SKG, SGD, PGG, PVA motifs were present in MAD20 and denoted as 5, 6, 7, 8, 5*, 5^#^ and 6*, respectively. Each letter in the tri-peptide motifs represents an amino acid

**Fig. 4:**
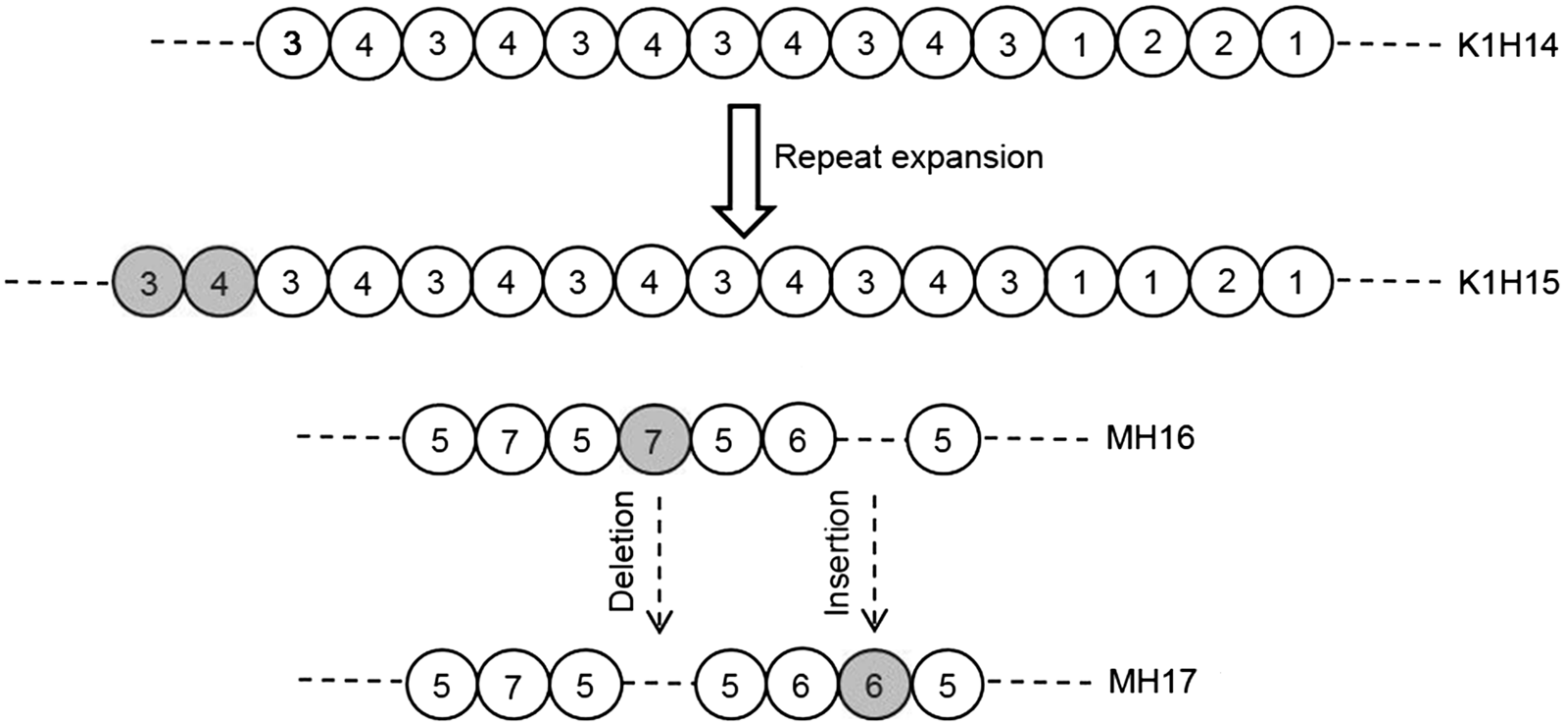
Possible mechanisms leading to allelic variability of *msp1* block 2. Repeat expansion and insertion/deletion are presumably responsible for generating K1H15 and MH17 from K1H14 to MH16, respectively

To refine the analysis further, K1 and MAD20 families were classified into multiple sub types according to the copy number and arrangement of tri-peptide motifs present. The parasite population of Chhattisgarh and West Bengal differed remarkably with respect to the distribution and frequency of sub-alleles (Fig. 3). Overall bin sizes of indel subtypes under K1 and MAD20 families were 16 (15 in Chhattisgarh and 1 in West Bengal) and 24 (17 in Chhattisgarh and 11 in West Bengal), respectively. Of the 6 distinct tri-peptide motifs observed in K1, four (SGT, SGP, SAQ and SGA, coded as 1–4) were previously reported, while two rare motifs namely STQ (conversion of **G**CT to **A**CT codon resulting in A to T substitution) and SAR (conversion of C**A**A to C**G**A codon resulting in Q to R substitution) were derived from SAQ to detected in two mild malaria patients having multiclonal infections. The members of MAD20 allele family were represented by four previously reported tri-peptide motifs such as SGG, SVA, SVT, and SKG (coded as 5–8) [45]. Three rare motifs including SGD (G**G**T > G**A**T), PGG (**T**CA > **C**CA), PVA (**T**CA > **C**CA), coded by 5*, 5^#^, 6*, respectively were also detected in Chhattisgarh population (Fig. 3 and Additional file 2: Table S2). Phylogenetic trees constructed based on tri-peptide copy number variation of K1 and MAD20 families in Chhattisgarh revealed a characteristic pattern of evolutionary relationship (Fig. 3). For instance, K1H15 (repeat motif = **34**343434343431221) seemed to be originated from K1H14 (repeat motif = 343434343431221) by repeat expansion of SAQ-SGA tri-peptide (Figs. 3, 4). On the other hand, MH17 (repeat motif = 5755665) seemed to be derived from MH16 (repeat motif = 575**7**565) through deletion of one SVT and insertion of one SVA motif (Fig. 4).

### Genetic diversity of *msp1* based on SNVs

To identify the footprints of genetic and population level forces shaping the *msp1* genetic diversity, multiple sequence alignment was performed using reads covering the regions flanking repeat expanse of block 2. Genomic region encompassing 64–120 amino acid residues in K1 and 81–131 residues in MAD20 were excluded from this analysis (Fig. 1) [29]. Since RO33 family lacked any indel variations, the complete sequence reads representing RO33 allele was available for identification of single nucleotide changes. All three allele families from Chhattisgarh parasite sequences harboured extensive sequence variation in the non-repetitive part of block 2. In contrast, West Bengal parasite population harbored variations only in the sequences belonging to MAD20 family. This was reflected in the nucleotide diversity estimates. For example, mean pairwise mismatches (k) for alleles belonging to K1, MAD20 and R033 in Chhattisgarh were 1.198, 6.414, 5.156, respectively; while those estimated for West Bengal sequences were 0, 6.104 and 0, respectively (Table 1). In Chhattisgarh samples nucleotide substitutions were distributed to both upstream and downstream regions flanking the tri-peptide motifs of K1 and MAD20 whereas in West Bengal population SNVs were clustered only in the region downstream to repeat motifs of MAD20. Finally, most variants found in K1 and MAD20 allelic background in Chhattisgarh samples were rare in frequency as evidenced by the negative Tajima’s D statistic (K1: − 2.536, MAD20: − 1.360) and statistically significant Fu & Li’s D* and F* estimates (K1: − 4.523 and − 4.574, MAD20: − 3.804 and − 3.492, respectively). In contrast, all the segregating sites found in MAD20 group in West Bengal were of intermediate frequency resulting in a positive Tajima’s D (1.305) as well as positive Fu & Li’s D* and F* indices (1.657 and 1.820), suggesting a signature of diversifying selection (Table 1).

**Table 1:** Genetic diversity parameters estimated for regions encompassing MSP1 block 2 and MSP1-19 in two Indian *P. falciparum* populations

Genomic region	Area	Sample size	Allele	No. of genotype	S (P)	k	π ± SD	θw ± SD	Rm	Tajima’s D	Fu and Li’s D*; F*
MSP1 block 2	CG	41	K1	30	17 (0)	1.198	0.004 ± 0.001	0.015 ± 0.004	0	− 2.536*	− 4.523*; − 4.574*
			MAD20	45	46 (14)	6.414	0.02 ± 0.003	0.039 ± 0.006	2	− 1.36	− 3.804*; − 3.492*
			RO33	10	12 (9)	5.156	0.015 ± 0.002	0.012 ± 0.004	1	0.973	0.540; 0.73
	WB	57	K1	7	–	–	–	–	–	–	–
			MAD20	45	18 (18)	6.104	0.023 ± 0.003	0.015 ± 0.004	1	1.305	1.657*; 1.82*
			RO33	5	–	–	–	–	–	–	–
MSP1-19	CG	19	–	–	7 (5)	1.45	0.005 ± 0.002	0.007 ± 0.003	1	− 0.921	0.07; − 0.24
	WB	30	–	–	4	1.363	0.005 ± 0.001	0.004 ± 0.002	0	0.899	1.058; 1.174

CG, Chhattisgarh; WB, West Bengal; S, Seggregating site; P, Parsimony informative sites; k, Average Number of Pairwise Differences; π, pairwise nucleotide diversity; θw, Watterson’s θ; Rm, minimum number of recombination events

* Indicates p < 0.05

Unlike *msp1* block 2, sequences encoding MSP1-19 showed relatively conserved genetic configuration as reflected by the low nucleotide diversity estimates namely θ, π and k (Table 1). Four non-synonymous substitutions at amino acid positions 1691 (T > K), 1700 (S > N), 1701 (R > G) and 1716 (L > F) were shared between two study sites whereas three additional rare variants were recorded only in Chhattisgarh samples. Interestingly, one of these rare mutations at position 4998 bp (C > T) altered glutamine (CAA) at 1666 to a stop codon (TAA) in one Chhattisgarh isolate [46]. This was presumably tolerated by the presence of another rare mutation (5000A > T) in the same patient. The remaining rare variant corresponded to a synonymous change.

### Comparison of sequence diversity among geographically diverse *P. falciparum* populations

**Fig. 5:**
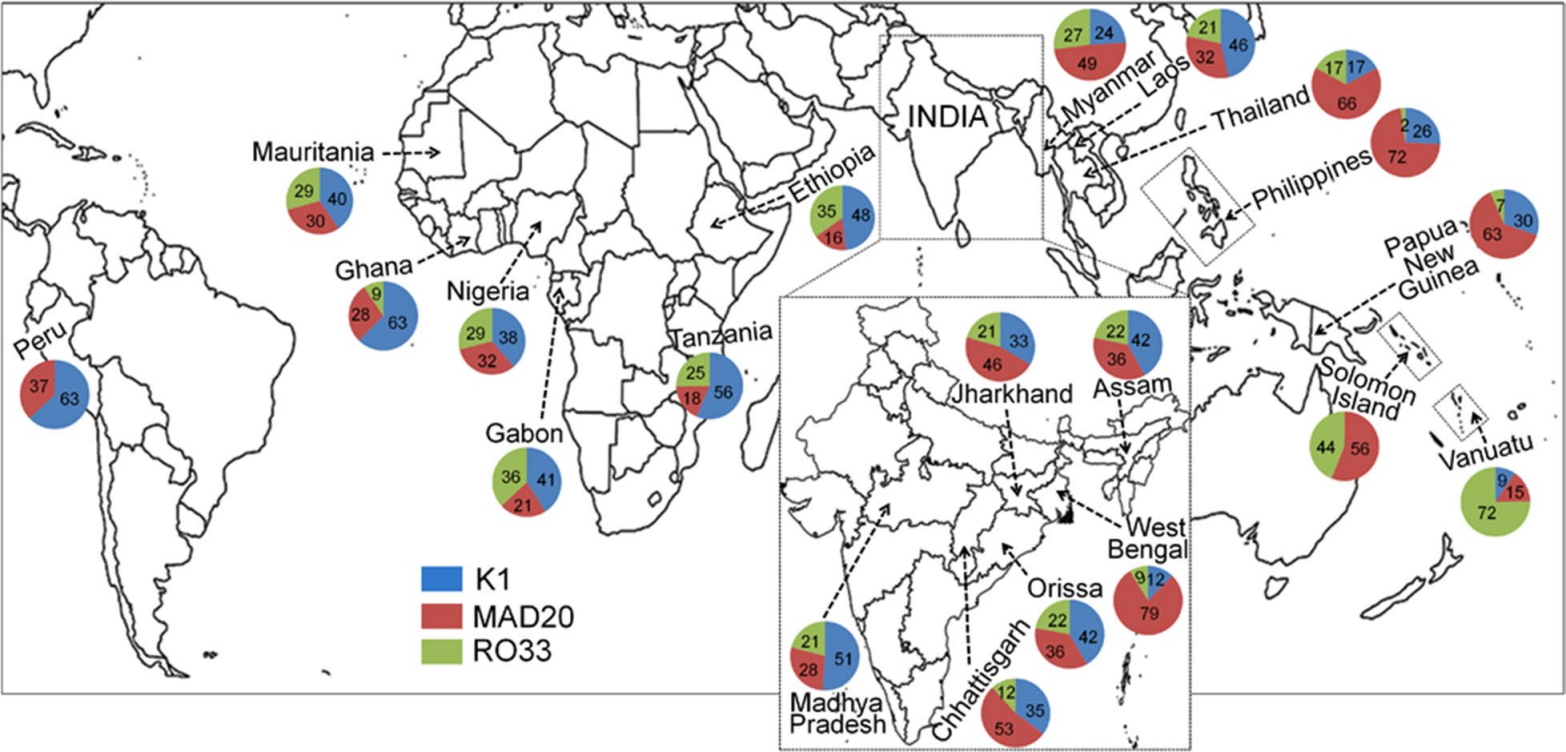
Worldwide distribution of *P. falciparum msp1* block 2 alleles. Frequencies of K1, MAD20 and RO33 in different geographical regions. Proportion of each allele in a certain parasite population was shown using pi diagram

To understand the pattern of genetic differentiation with respect to geographical distance among Indian parasite sub-populations and those present in other malaria endemic countries, *msp1* sequence data were retrieved from public databases [GenBank accession numbers: JF460898–JF460938, AB502443–AB502513, AB502514–AB502545, AB502546–AB502586, AB502587–AB502628, AB715434, AB502629–AB502704, AB502705–AB502745, AB715435–AB715519] [14, 47–57]. Except for the sub-populations from Assam and Orissa, all other pairwise comparisons in Indian isolates displayed statistically significant (p < 0.05) fixation indices (Table 2). Comparison of average allele frequencies of K1, MAD20 and RO33 in Indian sub-populations with that observed in other countries resulted in significant F_ST_ estimates for all pairwise tests (Additional file 3: Table S3). An analysis of frequency spectra of allele families indicated an overall prevalence of K1 and MAD20 in South East Asia, excepting Myanmar and Vanuatu. Abundance of RO33 was comparatively higher in African *P. falciparum* populations while it was absent in Peruvian Amazon of South America (Fig. 5). In summary, all inter population assessments indicated the existence of a strong local structure in the *P. falciparum* populations.

**Table 2:** Pairwise F_ST_ based on *msp1* block 2 allele frequencies in Indian *P. falciparum* sub populations

	Chhattisgarh	West Bengal	Jharkhand	Orissa	Madhya Pradesh
Chhattisgarh					
West Bengal	0.105*				
Jharkhand	0.011*	0.129*			
Orissa	0.037*	0.218*	0.013*		
Madhya Pradesh	0.077*	0.278*	0.048*	0.012*	
Assam	0.035*	0.215*	0.012*	0	0.012*

* Indicates p < 0.05

### Assessment of antigenic organization of observed MSP1 block 2 and MSP1-19 alleles

The next section examined how this extreme genetic variability of MSP1 block 2 may influence its antigenic potential. Numbers of variants subjected to linear epitope mapping were 16, 24 and 3 for K1, MAD20 and RO33, respectively (Additional file 4: Table S4). Epitope evaluation was initially conducted using the threshold score of 0.35. This revealed that every residue of K1 and RO33 and those located in an internal stretch of MAD20 could potentially be incorporated as an epitope. Prediction of epitopes was then repeated using the stringent threshold score of 1.30. Two independent stretches of amino acids with variable lengths and sequences emerged as potential epitopes for each of K1 (13–66 residues) and MAD20 (8–57 residues) variants (Additional files 5 and 6: Figures S1, S2). Numbers of unique epitope predicted for K1, MAD20 and R033 were 18, 31 and 1, respectively (Table 3). Of the 18 different K1 epitopes, SNTSSGASPPADA was present in 84% (31 out of 37) parasite field isolates. Among MAD20 epitopes, GGSGNSRRTNPSDNSSDSDAK was present in 98.8% (89 out of 90) of parasites either as an independent motif (epitope #3) or as the part of a larger epitope (epitope #4, 5, 6, 7, 8, 11, 13, 16, 31) (Table 3). A single epitope, QSAKNPPGATVPSGTAS, with slightly variable scores represented all 3 RO33 alleles observed among 11 isolates. In summary, each block 2 family could be represented by a unique antigenic determinant and 94.9% (131 of 138) of parasites was represented by 3 predominant epitopes. Average epitope score of block 2 peptides was the highest for K1 (2.076 ± 0.145) followed by R033 (1.872 ± 0.007) and MAD20 (1.749 ± 0.129).

**Table 3:** Probable B cell epitopes of MSP1 block 2 and MSP1-19 variants

#	Epitope sequence	Length (AA)	Subtype	Mean residue score
Potential B cell epitopes for K1				
1	ASAQSGTSGTSGTSAQSGTSGTSGTSAQSGT SGTSGTSGTSGPSGPSGPSGPSGPSGTSPSSR	63	K1H6	2.226
2	ASAQSGTSGTSGTSGPSGPSGTSPSSR	27	K1H13	2.216
3	ASAQSGTSGTSGPSGTSGPSGTSPSSR	27	K1H12	2.216
4	ASAQSGTSGTSGTSGTSGPSGPSGTSPSSR	30	K1H10	2.201
5	SARSGTSGTSGTSGPSGPSGTSPSSR	26	K1H14	2.200
6	ASAQSGTSGTSGTSAQSGTSGTSGTSGTSGTS GPSGTSGTSGTSGTSGTSGPSGPSGPSGTSPSSR	66	K1H7	2.199
7	ASAQSGTSGTSGTSGTSGTSGTSGPSGPSGTSPSSR	36	K1H9	2.179
8	SNTSSGTSPPADA	13	K1H13	2.173
9	ASAQSGTSGTSGTSGTSGTSGTSGTSGTSGTSGPSGPSGTSPSSR	45	K1H8	2.157
10	ASAQSGTSGTSGTSAQSGTSGTSGTSGTSGTSGPSGPSGTSPSSR	45	K1H15	2.111
11	*SNTSSGASPPADA*	13	K1H1, K1H2, K1H3, K1H4, K1H5, K1H6, K1H8, K1H9, K1H10, K1H11, K1H14, K1H15, K1H16	2.092
12	ASAQSGTSGTSGTSAQSGTSGTSAQSGTSGTS GTSGPSGPSGTSPSSR	48	K1H16	2.065
13	ASTQSGASAQSGASAQSGASAQSGTSGTSGPSGPSGTSPSSR	42	K1H5	1.962
14	ASAQSGASAQSGASAQSGASAQSGTSGTSGPSGPSGTSPSSR	42	K1H4	1.944
15	ASAQSGTSGTSGTSAQSGTSGTSPSSR	27	K1H11	1.890
16	ASAQSGASAQSGASAQSGASAQSGASAQSGTS GPSGPSGTSPSSR	45	K1H3	1.883
17	ASAQSGASAQSGASAQSGASAQSGASAQSGAS AQSGTSGPSGPSGTSPSSR	51	K1H2	1.845
18	ASAQSGASAQSGASAQSGASAQSGASAQSGAS AQSGASAQSGTSGPSGPSGTSPSSR	57	K1H1	1.815
Potential B cell epitopes for MAD20				
1	PDAANPSDNSSDSDAK	16	MH12	2.025
2	AVTTSTPGSKGSGGSVA SGGSGGSGGSGGSGGP	33	MH9	1.961
3	*GGSGNSRRTNPSDNSSDSDAK*	21	MH3, MH9, MH11, MH13, MH15, MH17, MH24	1.908
4	AVTTSTPGSGGSVTSGGSGGSGGSGG SVAS*GGSGNSRRTNPSDNSSDSDAK*	51	MH23	1.896
5	VAS*GGSGNSRRTNPSDNSSDSDAK*	24	MH1, MH5, MH6, MH7, MH10, MH19, MH21, MH22	1.872
6	GGSGGSVAS*GGSGNSRRTNPSDNSSDSDAK*	30	MH14	1.869
7	GSVAS*GGSGNSRRTNPSDNSSDSDAK*	26	MH4	1.855
8	SVAS*GGSGNSRRTNPSDNSSDSDAK*	25	MH16	1.853
9	AVTTSTPGSKGSVTSGGSGGSGGSGGSVA	29	MH22	1.829
10	AVTTSTPGSKGSGGSVASGGSGGSGG	26	MH11	1.812
11	AVTTSTPGSKGSVTSGGSGGSGGSVASGG SGGSVAS*GGSGNSRRTNPSDNSSDSDAK*	57	MH20	1.797
12	AVTTSTPGSKGSVTSGG SGGSGGSGATVPSGTAS	31	MH12	1.797
13	AVTTSTPGSKGSVAS*GGS* *GNSRRTNPSDNSSDSDAK*	36	MH18	1.794
14	AVTTSTPGSKGSVTSGGSGGSGGSVA	26	MH2	1.760
15	AVTTSTPGSGGSVT	14	MH24	1.760
16	AVTTSTPGSKGSVTSGGSGGSVASGGSG GSVAS*GGSGNSRRTNPSDNSSDSDAK*	54	MH8	1.760
17	AVTTSTPGSKGSGGSVA	17	MH10	1.758
18	AVTTSTPGSKGSVTSGGSGGSGGSVA	26	MH1, MH3	1.755
19	AVTTSTPGSGGSVTSGGSGGSVTSGGSGG	29	MH13	1.746
20	AVTTSTPGSGGSVTSGGSGG	20	MH14, MH15, MH17	1.745
21	AVTTSTPGSKGSVTSGG SGGSGGSVASGGSGG	32	MH6	1.733
22	GGTAVTTSTPGSGGSVT	17	MH16	1.710
23	AVTTSTPGSKGSVTSGGSGGSVASGGSGGSVASGGSGGSVA	41	MH7	1.681
24	AVTTSTPGSKGSVTSGGSGGSVASGGSGG	29	MH4, MH5, MH6	1.665
25	SVTSGGSGG	9	MH24	1.649
26	VASGGSGGSVASGGSGGSVA	20	MH19	1.615
27	VASGGSGGSVA	11	MH10	1.598
28	VASGGSGG	8	MH1, MH3, MH22	1.583
29	SVASGGSGG	9	MH2	1.567
30	AVTTSTPGSVA	11	MH19	1.499
31	VAS*GGSGNSRRTNPSDNSSDSDAK*	24	MH2	1.458
Potential B cell epitopes for RO33				
1	*QSAKNPPGATVPSGTAS*	17	Rhap1	1.880
			Rhap2	1.869
			Rhap3	1.867
Potential B cell epitope on MSP1-19				
1	TEEDSGSN	8	Mhap 1, Mhap 4	1.734
2	TEEDSGS	7	Mhap 2, Mhap 3	1.676
3	VENPNPTCNENNGGC	15	Mhap 3	1.552
4	*CVENPNPTCNENNGGC*	16	Mhap 2	1.539
			Mhap 1, Mhap 4	1.537

Epitopes were defined using the threshold score of 1.30. Predominant epitopes were marked in italics

**Fig. 6:**
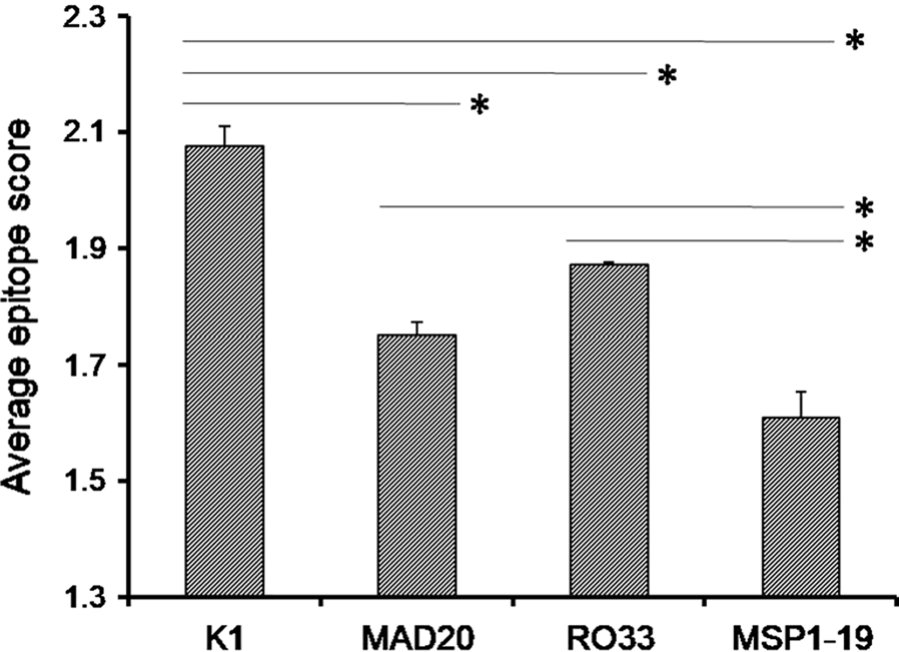
Comparison of average epitope scores of MSP1 block 2 and MSP1-19 peptides in India. Epitopes were predicted based on a threshold score of 1.3. Asterisk indicates p < 0.05 in two-tailed Student’s t test

Similar analysis was conducted on 4 haplotypes of MSP1-19 and it revealed that average epitope score for this relatively conserved segment of MSP1 was significantly (< 0.05) lower (1.608 ± 0.091) than any of the probable block 2 antigens as per Student’s t test (Fig. 6).

## Discussion

The significant decline of global malaria burden achieved in the last 15 years is mainly attributed to the use of insecticide-treated nets and implementation of artemisinin-based combination therapy (ACT) [58]. Two factors that still hinder the progress of malaria control include the emergence of drug-resistant parasite strains and development of vectors resistant to insecticide [59]. Development of a malaria vaccine would be an additional arsenal to the existing tools for malaria control. One of the challenges in developing malaria vaccines is the extensive genetic diversity of parasite antigens that are vaccine targets. Individuals living in areas of high transmission intensity are often simultaneously infected by multiple parasite genotypes [60]. It is, therefore, important to characterize the level of parasite genetic variation in diverse geographical locations to identify the prevailing parasite strains. To this end, this article provides a comprehensive description of *P. falciparum* diversity for two most important immunogenic segments of MSP1 in disparate malaria affected regions of India. In addition it makes an attempt to correlate the variability of the protein sequences with its antigenic properties.

MSP1 is one of the prime candidates for the development of malaria blood stage vaccine and it serves as a suitable marker for the identification of genetically distinct *P. falciparum* populations [50]. Analysis of *msp1* block 2 reveals predominance of MAD20 in both geographical regions, studied. Similar prevalence of MAD20 was observed in studies conducted in Baikunthpur and Madhya Pradesh, two neighboring regions and those from Philippines, Papua New Guinea and Myanmar [28, 48, 49, 54]. In contrast, a higher frequency of K1 has been reported from Orissa, Madhya Pradesh, Assam in India and Mauritania and Uganda [47, 48, 56, 61]. It is important to note in this context that several of these studies including those conducted on Indian sub-populations used PCR followed by hybridization with allele-specific probes to capture the allelic diversity of block 2. Since this technique relies on size discrimination of products ranging from 400 to 600 bp, it is possible that some unique *msp1* alleles remain indistinguishable because of their proximity of sizes [62].

The present study recovers a total number of 33 different indel parasite alleles based on the sequence diversity of block 2 in Chhattisgarh whereas the parasite sub-population from West Bengal harbours 13 indel sub-alleles. Only 10.42% of allele pools are shared between the hyper- and hypoendemic states of Chhattisgarh and West Bengal, respectively. This high level of genotypic diversification and low level of gene migration among Indian parasite sub-populations have been supported by statistically significant F_ST_ estimates. As expected, Ambikapur parasite population shares 34% and 28% of indel variants with those from neighbouring regions of Baikunthpur and Madhya Pradesh, respectively [28, 48].

Point mutation and repeat instability due to recombination are the major factors responsible for variability of K1 and MAD20. For instance, K1H7 (repeat motif = 3111111221) seems to be originated from K1H6 (repeat motif = 31111221) by repeat expansion of STG tri-peptide in Chhattisgarh. Such extensive variability has presumably been evolved as an immune evasion mechanism by the parasite in which protective immune response mounted by the host has favoured diversifying selection of block 2 [22]. A finer evaluation of *msp1* repeat organization in Chhattisgarh data suggests that K1 alleles may be broadly classified in two sub-families (starting with code: 3/3^#^11… or 3/3*434343…) while MAD20 family exhibits three sub-groups (starting with code: 875…, or 8565…, or 575…). The complexity of Chhattisgarh parasite population is exemplified by the observation that 39.02% patients suffered from multi-genotypic infections (ranging from 2 to 7). This statistics is comparable with that of Baikunthpur where 37% of the samples carried polyclonal infections with a MOI of 1.67 [28]. This data and that of others indicate a possible positive association between MOI and endemicity of *P. falciparum* [63–66]. Nevertheless, this correlation may not be an absolute one as MOI of *P. falciparum* ranges from 1.00 to 2.70 in few hypoendemic regions of Southeast Asia [67, 68]. A very high MOI of 3 ± 2.24 detected in the age group ≤ 18, is suggestive of a weaker immunity of younger people. A negative correlation between MOI and disease severity in Chhattisgarh (mild malaria: 2.33 ± 1.78; severe malaria: 1.57 ± 1.02) is another notable observation.

To identify footprints of genetic and population level forces, sequences adjacent to the repeat expanse of block 2 and genomic region covering MSP1-19 are scanned for SNVs. MSP1-19 displays limited sequence heterogeneity. Of the ten MSP1-19 allelic forms reported globally, Indian field isolates harbor 4 non-synonymous substitutions suggesting the probable influence of purifying selection shaping the diversity of this functionally important portion of *msp1* gene [46]. Thus, the present study demonstrates that different kinds of selection forces shape the complex genetic landscape of MSP1.

Of the different MSP1 segments, most vaccine studies focus on the conserved C-terminal region of MSP1-19, although the block 2 region also elicits functionally protective immune responses and is associated with reduced risk of malaria [7, 22, 23, 69–71]. The immune responses to MSP1-19 and block 2 mediated predominantly by IgG1 and IgG3 subclasses, respectively [72, 73]. In vitro assays with purified IgG3 from malaria immune individuals have established the functional superiority of IgG3 as an inhibitor of parasite growth [7, 73, 74]. However, the extensive polymorphic nature of block 2 is a potential challenge.

To this end, the present study elaborates the antigenic properties of MSP1 block 2 and MSP1-19 by evaluating their probable antigen conformations and potencies using BepiPred. Forty-three MSP1 block 2 variants observed in 138 *P. falciparum* field isolates generate 50 unique linear B-cell epitopes. However, 94.9% (131 of 138) of parasites may be represented by only 3 conserved block 2 epitopes. In addition, the average epitope score for each of these three representative block 2 antigens are noticeably higher compared to that of MSP1-19. A polyvalent recombinant protein incorporating these three block 2 epitopes together with a sequence from MSP1 block 1, has been shown to induce high titre antibodies against a wide range of allelic types of *P. falciparum* field isolates [75]. On the contrary, a recent comparative analysis suggests that the global MSP1-42 population is not as tightly conserved as it has been thought previously [76]. This reinforces the importance of MSP1 block 2 modules as effective blood-stage malaria vaccine.

One drawback of the current study is that due to lack of required crystallographic structure of block 2, the analysis remains limited to evaluation of linear B-cell epitopes instead of conformational epitopes which are believed to be better suited for most biomedical applications. However, this may also be borne in mind that predicted conformation-based antigenic determinants may not always be immunologically functional and biochemically verifiable. Prediction of B-cell linear epitopes has often been served as an alternative procedure for proteins that are not structurally well characterized [77].

## Conclusion

Taken together, the present study identifies a high level of genetic differentiation between the parasite populations of Chhattisgarh and West Bengal which arises presumably due to lack of gene flow and difference in malaria transmission intensities. It also indicates that an opposing pattern of natural selection may operate on *msp1* block 2 and MSP1-19. The most remarkable finding of the current study, nevertheless, is the presence of a limited number of conserved epitopes representing the MSP1 block 2 despite its extensive genetic diversity. This kindles the possibility of vaccine development based on this immunologically active merozoite segment.

## Additional files

**Additional file 1: Table S1.** Selected thresholds and corresponding sensitivity & specificity estimates of BepiPred.

**Additional file 2: Table S2.** Tripeptide motifs and their corresponding nucleotide sequences for K1 and MAD20.

**Additional file 3: Table S3.** Pairwise F_ST_ estimated using *msp1* block 2 allele frequencies in different *P. falciparum* populations worldwide.

**Additional file 4: Table S4.** MSP1 block 2 and -19 alleles and NCBI accession numbers.

**Additional file 5: Figure S1.** Distribution of epitope scores in different K1 block 2 sub-alleles. N, N-terminal; C, C-terminal.

**Additional file 6: Figure S2.** Distribution of epitope scores in different MAD20 block 2 sub-alleles. N, N-terminal; C, C-terminal.

## Abbreviations

MOI: multiplicity of infection
MSP1: merozoite surface protein 1
PCR: polymerase chain reaction
SNV: single nucleotide variation
WHO: World Health Organization
