# Supplementary material for: Diversity analysis of MSP1 identifies conserved epitope organization in block 2 amidst high sequence variability in Indian Plasmodium falciparum isolates
Source: Malar J. 2018 Dec 3;17:447. doi: 10.1186/s12936-018-2592-y (PMC6276175; doi:10.1186/s12936-018-2592-y)
Supplement: Supplementary file 6 — Additional file 6: Figure S2. Distribution of epitope scores in different MAD20 block 2 sub-alleles. N, N-terminal; C, C-terminal. [file 12936_2018_2592_MOESM6_ESM.docx]

**
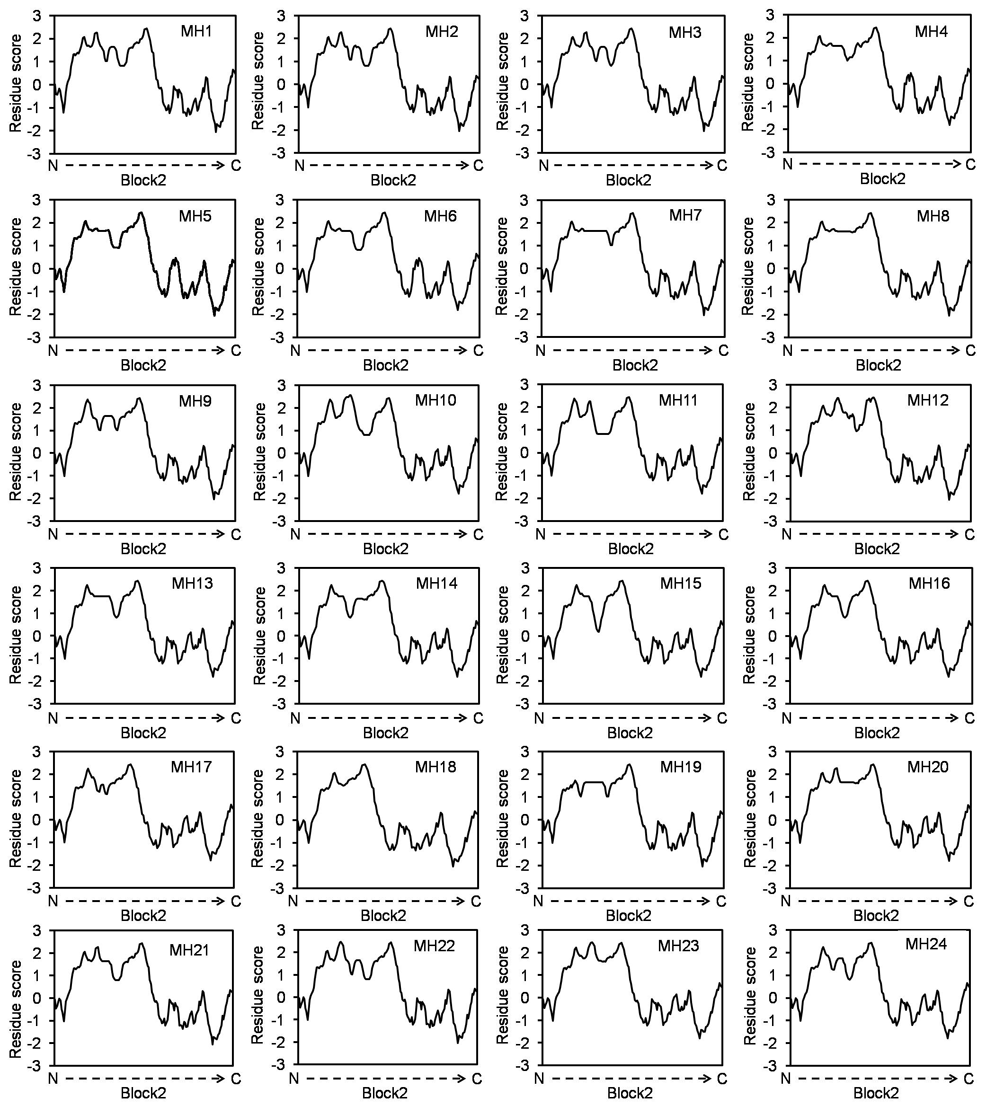
Additional file 6: Fig. S2.** Distribution of epitope scores in different MAD20 block 2 sub-alleles. N, N-terminal; C, C-terminal.
